# A Dynamic Foundation: Aberrations of Sleep Architecture and Its Association With Clinical and Sub-clinical Psychopathology

**DOI:** 10.7759/cureus.55262

**Published:** 2024-02-29

**Authors:** Richard C Todd

**Affiliations:** 1 Psychiatry, International European University School of Medicine, Kiev, UKR; 2 Research, Trinity Medical Sciences University, Warner Robins, USA

**Keywords:** hallucinations, hypnagogic, hypnopompic, narcolepsy, neurology, psychiatry, psychosis, schizophrenia, sleep, sleep-paralysis

## Abstract

This investigation centered on the hypnagogic and hypnopompic wake-sleep/sleep-wake transition states and the associated exploration of hypnagogic and hypnopompic experiences (HHEs), and sleep paralysis (SP) on psychiatric exacerbation and paradoxical masking. The study aims to discern causality by examining how these sleep-related experiences may contribute to the emergence or exacerbation of psychiatric and neurodegenerative conditions, particularly, pertaining to the clinical or sub-clinical demographic of Schizotypal Personality Disorder (STPD), Mood Disorders, Schizophrenia, Post-Traumatic Stress Disorder (PTSD), Generalized Anxiety Disorder (GAD), Narcolepsy, Panic Disorder, specific phobias, or heightened psychotic sensitivity. Methodologically, this study employed a comprehensive literature review, drawing from a range of studies across sleep medicine, psychiatry, and psychology, utilizing PubMed-indexed peer-reviewed scientific literature, sourcing from academic institutions, Google Scholar, and open-access publications. This interdisciplinary approach allowed for a nuanced and systematic understanding of the potential links between specific sleep-wake/wake-sleep aberrations and their masking or exacerbation of clinical/sub-clinical psychiatric symptomatology in this particular demographic. Insights gained from the outcome of this study hold promise for advancing understanding of the interrelationship between sleep neurobiology and psychiatric disorders. Additionally, the findings may inform targeted therapeutic interventions tailored to mitigate the impact of sleep-wake disruptions on vulnerable populations. The overarching objective is to bridge current gaps in knowledge, cultivating a more profound understanding with direct implications for both clinical practice and ongoing research endeavors. The study outcomes provide an intriguing understanding of the complex relationship between sleep neurobiology and psychiatric disorders, paving the way for targeted therapeutic interventions to alleviate the effects of sleep-wake disruptions, and addressing critical gaps in knowledge with direct implications for clinical practice and ongoing research.

## Introduction and background

Sleep, an essential physiological process, plays a pivotal role in maintaining overall health and cognitive function. Recent scientific inquiries have delved into the intricate interplay between sleep architecture and mental health, unraveling a profound connection that extends beyond conventional understanding. This study embarked on a comprehensive exploration of the relationship between maladaptive sleep architecture and its potential influence on psychiatric manifestations, particularly in individuals displaying clinical or subclinical susceptibility to mental health disorders. This inquiry focused on patients who exhibited heightened sensitivity to psychiatric pathology and encompassing psychiatric pathology, such as Schizotypal Personality Disorder (STPD), Mood Disorders, Schizophrenia, Post-Traumatic Stress Disorder (PTSD), Generalized Anxiety Disorder (GAD), Narcolepsy, Panic Disorder, specific phobias, and heightened psychotic sensitivity. By homing in on this specific demographic, this study aimed to unravel the putative relationship between sleep aberrations and the emergence, masking, or exacerbation of psychiatric and neurodegenerative conditions. At the heart of this investigation lies the hypnagogic and hypnopompic states, pivotal phases in the sleep-wake transition that offer a gateway to unique experiences, such as hypnagogic and hypnopompic experiences (HHEs) and sleep paralysis (SP) [1]. These phenomena, often overlooked in conventional psychiatric assessments, warrant meticulous examination to discern their potential role in their complex tapestry of psychiatric symptomatology.

The insights gleaned from this study hold promise for advancing our comprehension of the interrelationship between sleep neurobiology and psychiatric disorders. Beyond contributing to the theoretical framework, this study’s findings may pave the way for targeted therapeutic interventions, specifically tailored to mitigate the impact of sleep-wake disruptions on vulnerable populations. By addressing current gaps in knowledge, our exploration aims to cultivate a profound understanding with direct implications for both clinical practice and ongoing research endeavors in the realm of sleep and mental health. Sleep paralysis (SP) characterizes a transitional dissociative state commonly experienced during the transition from wakefulness to sleep or vice versa [2]. During SP episodes, individuals face the unusual phenomenon of muscle atonia, rendering them immobile while remaining conscious [2]. Often, these episodes involve hypnopompic and hypnagogic hallucinations, spanning visual, tactile, kinesthetic, auditory, and occasionally olfactory experiences [3-6].

The hallucinatory experiences during SP fall into three distinct categories: the "intruder," marked by a sense of a threatening or hostile presence, fear, and auditory or visual hallucinations, often manifesting as a shadow or dark form; the "incubus," characterized by sensations of tightness or pain in the chest, breathing difficulties, and occasional visual hallucinations, such as a figure sitting on the chest; and vestibular-motor hallucinations, involving peculiar bodily sensations like levitation, spinning, autoscopy, and out-of-body experiences (OBEs) [1,4,5,7,8].

Psychosomatic sensations frequently accompany SP episodes, including feelings of chest pressure, breathing difficulty, suffocation, heart palpitations, sweating, and nausea [9]. These experiences are commonly associated with heightened fear or a fear of imminent mortality [1,3,4]. SP can occur independently, termed as isolated sleep paralysis (ISP), or as a manifestation of other medical conditions, such as narcolepsy and seizure disorders [10]. When episodes of ISP recur, they are classified as a distinct diagnostic sleep-wake disorder known as recurrent isolated sleep paralysis (RISP), falling under the category of rapid eye movement (REM) sleep phase parasomnia [11].

These experiences can be highly distressing, potentially eliciting fear, anxiety, unconventional descriptions, and explanations [11]. Consequently, individuals undergoing such encounters could be susceptible to psychiatric diagnoses [12-14]. Although earlier investigations primarily focused on epidemiological surveys within the general population [15-17], there is an emerging research effort probing SP within patients having diverse diagnoses.

There is quite a significant overlap regarding specific symptoms of psychiatric disorders and the often-overlooked phenomena of HHEs and SP. And with regard to this, the misdiagnosis of patients with HHE hallucinations and SP as having schizophrenia has been identified and highlighted [18]. Primarily, this is due to the similarity in hallucinations and the overlapping age of onset [19]. Furthermore, patients with narcolepsy may experience psychosis in the form of associated HHEs and SP [19-22]. Consequently, distinguishing between psychosis in schizophrenia and the psychotic form of narcolepsy is crucial. Failure to identify narcolepsy often leads to the misclassification of patients as being refractory to standard schizophrenia treatments [23]. This is where a new differential can be identified to appropriately treat the narcolepsy, after revisiting and gathering appropriate patient history regarding sleep-related changes. Identifying the misdiagnosed narcolepsy with psychotic features is crucial, as it leads to a resolution of psychotic signs, not through antipsychotics, but with psychostimulants [24]. Previous research on patients diagnosed with schizophrenia also indicated that a subset, approximately 4-7%, could actually have an undiagnosed psychotic variant of narcolepsy [25,26].

This intricate interplay between hallucinations, misdiagnosis, and the development of psychosis underscores the necessity for a nuanced approach in clinical assessments, and highlights the need for accurate differentiation between HHEs and SP seen in narcolepsy-related phenomena, schizophrenia, and other psychotic manifestations of reality, as pertaining to psychiatric experience.

According to a case report published in Sage, “…A 25-year-old black South African woman developed paranoid beliefs and a sad and anxious mood in the wake of her first experience of sleep paralysis and hypnic hallucinations. She had no history of other sleep-related events” and it was concluded that, “…Acute, nocturnal-onset, first-time psychopathology warrants inquiry for sleep paralysis and hypnic hallucinations…” [12]. This patient, as a result of their experience regarding HHEs and SP, began manifesting differential criteria for psychopathology [12]. It was only until further clarification regarding this typically benign parasomnia, that the patient’s acute paranoia, depression, and anxiety subsided without recurrence [12].

To further illustrate such causation/correlation, a University-led study (n = 100) was carried out in 2015 to examine the relationship between SP and anxiety symptoms among Egyptian college students, as well as possible post-traumatic stress disorder (PTSD), trait anxiety, and pathological worry [27]. Overall, 43% of the university participants reported at least one lifetime episode of SP, and 24% of those who reported at least one lifetime episode had experienced four or more episodes during the previous year [27]. Participants who had SP reported higher symptoms of PTSD, trait anxiety, and pathological worry. In addition, the experiencing of HHEs during SP, even after controlling for negative effect/s, was highly correlated with later symptoms of PTSD and trait anxiety [27].

Research carried out in 2018 by Grandner and colleagues, focusing on SP/HHEs experienced by athletes (n=189), found that the degree to which people reported such symptoms actually predicted the severity of depression symptoms, even after controlling for poor sleep and lack of sleep [13].

Consequently, the aim of this study was to discern causality by examining how these sleep-related experiences may contribute to the emergence or exacerbation of psychiatric and neurodegenerative conditions, particularly, pertaining to the clinical or sub-clinical demographic of Schizotypal Personality Disorder (STPD), Mood Disorders, Schizophrenia, Post-Traumatic Stress Disorder (PTSD), Generalized Anxiety Disorder (GAD), Narcolepsy, Panic Disorder, specific phobias, or heightened psychotic sensitivity.

## Review

Study design

A systematic review and meta-analysis was undertaken to examine the prevalence of maladaptive sleep architecture and its potential impact on psychiatric and neurological disorders, drawing upon existing epidemiological studies. Adhering to the latest Preferred Reporting Items for Systematic Reviews and Meta-Analyses (PRISMA) guidelines, this comprehensive review aimed to provide a rigorous and standardized assessment of the available evidence in the field [28].

Search strategy

An extensive literature search was conducted to identify studies examining the prevalence of HHEs and SP. The search, finalized on January 4, 2024, employed a combination of specific terms, including "sleep paralysis," "isolated sleep paralysis," "parasomnia not otherwise specified," "hypnagogic," "hypnopompic," and "parasomnia." Furthermore, this study expanded the search to encompass indirect references to sleep paralysis, such as "nighttime hallucinations," "nighttime paralysis," and "hallucinations upon awakening." To ensure completeness, backward searches were carried out to identify pertinent articles in the field.

Data sources and screening

**Figure 1:**
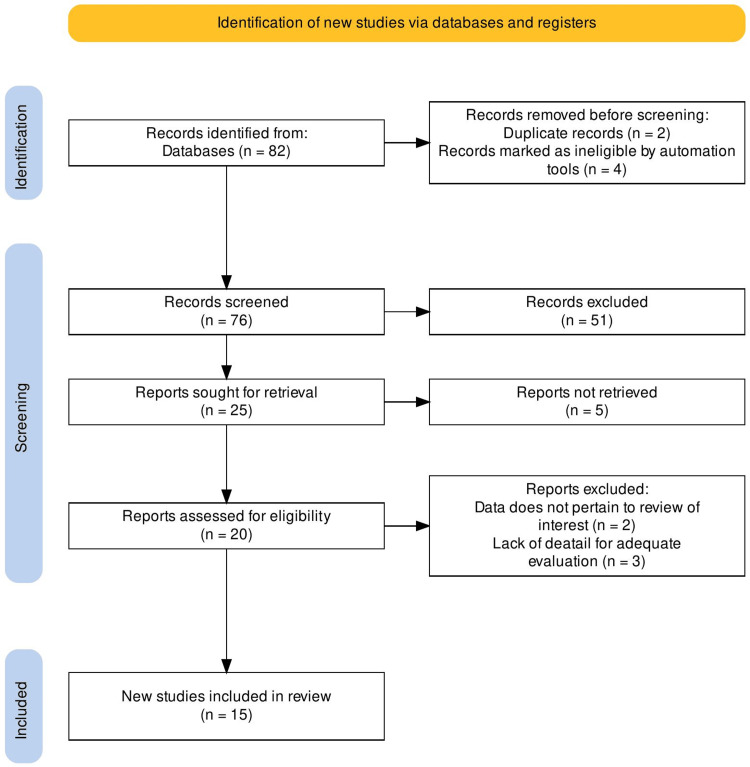
Overview of study screening workflow, according to PRISMA guidelines

The search spanned four resource models (PubMed, Google Scholar, academic institutions, and open-access peer-reviewed literature), from this study’s inception to January 2024. A single independent author meticulously reviewed the selected studies through the Rayyan online platform (Rayyan Systems, Inc., Cambridge, Massachusetts, United States). The inclusion criteria encompassed all populations, with no exclusions, based on age, gender, education, residency, or mental health state. In terms of study design, this systematic review incorporated observational studies, including cross-sectional, cohort, and case-control studies. Included were studies that examined SP and HHEs as either primary or secondary outcomes. This study was limited to only include studies conducted in English. Conversely, reviews, conference abstracts, and studies in languages other than English - without an available English version - were excluded. No additional restrictions were applied during the screening process (Figure 1). Overall, 82 report records were identified, 76 were screened, with 51 being excluded, as depicted in Figure 1 below. Subsequently, the remaining 25 reports were sought for retrieval, and a total of 15 reports were deemed to be eligible for inclusion within this systematic review (Figure 1 and Table 1).

**Table 1:** List of included relevant articles in systematic review concerning HHEs and SP correlation to psychiatric conditions. HHE: Hypnagogic and hypnopompic experience; SP: Sleep paralysis.

Authors	Title	Reference
Kishi et al.	Schizophrenia and narcolepsy: A review with a case report	[19]
Ohayon et al.	Hypnagogic and hypnopompic hallucinations: Pathological phenomena?	[25]
Douglass et al.	Schizophrenia, narcolepsy, and HLA-DR15, DQ6	[26]
Douglass et al.	Florid refractory schizophrenias that turn out to be treatable variants of HLA-associated narcolepsy	[23]
Gangdev	Relevance of Sleep Paralysis and Hypnic Hallucinations to Psychiatry	[12]
Jalal and Hinton	Sleep Paralysis Among Egyptian College Students: Association With Anxiety Symptoms (PTSD, Trait Anxiety, Pathological Worry)	[27]
Naumann and Daum	Narcolepsy: pathophysiology and neuropsychological changes	[20]
Burgess and Scammell	Narcolepsy: neural mechanisms of sleepiness and cataplexy	[21]
Shedler and Westen	Refining personality disorder diagnosis: integrating science and practice	[22]
Wrobel-Knybel et al.	Characteristics of Sleep Paralysis and Its Association with Anxiety Symptoms, Perceived Stress, PTSD, and Other Variables Related to Lifestyle in Selected High Stress Exposed Professions	[10]
Gangdev	Sleep paralysis accompanied by secondary psychiatric disturbance	[8]
Gangdev and Ramjee	Sleep paralysis presenting with reactive depression	[6]
Gangdev et al.	Isolated sleep paralysis and hypnic hallucinations in schizophrenia	[18]
Sandoiu and Collier	Sleep paralysis, hallucinations may raise depression risk in some people	[13]
Hany et al.	Schizophrenia	[14]

The following section describes the differing findings obtained from the included articles from this literature review, and highlights the varying correlations of HHEs and SP with psychiatric conditions.

Psychopathology affected by HHEs and SP, associated pathophysiology, and etiology

Narcolepsy is, undoubtedly, a complex sleep disorder involving genetic predisposition, abnormal neurotransmitter functioning/sensitivity, and abnormal immune modulation such as human leukocyte antigen (HLA) subtypes and abnormal hypocretin (orexin) neurotransmission, as found within this systematic review. These factors contribute to abnormalities in monoamine and acetylcholine synaptic transmissions, particularly within the pontine reticular activating system [20,21]. Understanding the multifaceted nature of narcolepsy is crucial for accurate diagnosis, as well as for addressing the various misconceptions surrounding the disorder among patients, parents, teachers, and healthcare professionals.

Schizotypal Personality Disorder (STPD) includes symptoms such as pronounced eccentricities in thought, perception, and behavior, including excessive social anxiety that persists despite familiarity, and encountering idiosyncratic perceptual experiences or bodily illusions [22]. Additionally, personality disorders have been associated with reduced levels of monoamine oxidase (MAO) and downstream effects on serotonin levels [29]. However, it is important to note that the current understanding of the relationships between anatomy, receptors, and neurotransmitters to personality disorders remains speculative. Further research is needed to elucidate these connections.

Due to its inherent complexity and diversity, the full understanding of the etiology and pathophysiological mechanisms of schizophrenia remains incomplete [14]. Schizophrenia may stem from abnormalities in various neurotransmitters, involving dopaminergic, serotonergic, and alpha-adrenergic hyperactivity or glutaminergic and GABA hypoactivity [30].

Genetics also plays a pivotal role, with a 46% concordance rate in monozygotic twins and a 40% risk if both parents are affected. Genes like neuregulin (NGR1), associated with glutamate signaling and brain development, dysbindin (DTNBP1), influencing glutamate release, and catecholamine O-methyl transferase (COMT) polymorphism, regulating dopamine function, have been implicated [14].

Several environmental factors have been identified as contributing to an elevated risk of developing schizophrenia. These factors include abnormal fetal development and low birth weight, gestational diabetes, pre-eclampsia, emergency cesarean section, and other birth complications. Additionally, maternal malnutrition and vitamin D deficiency have been associated with an increased risk. Winter births, linked to a 10% higher relative risk, and urban residence, which elevates the risk by 2 to 4%, further contribute to the complex interplay of environmental elements influencing the susceptibility to schizophrenia [14].

## Conclusions

This comprehensive exploration of the relationship between maladaptive sleep architecture (particularly, HHEs/SP), and psychiatric manifestations, has yielded compelling evidence, suggesting a correlation that warrants careful consideration in the clinical setting. Such findings underscore the significance of recognizing HHEs and SP as potential contributors to the exacerbation of pre-existing psychopathologies or the induction of misdiagnoses, particularly in cases of sub-clinical presentations. The overlap in symptoms between parasomnias and certain psychiatric disorders, as highlighted in this review, emphasizes the importance of including parasomnia in the first-time differentials for patients reporting HHEs and SP. Failure to consider parasomnia in the differential diagnosis may lead to misattributions of symptoms and potentially inappropriate treatment strategies. Specifically, this research underscores the need for heightened awareness in distinguishing between hallucinations associated with parasomnia and those indicative of serious psychiatric conditions such as schizophrenia. The misdiagnosis of narcolepsy-related parasomnia phenomena as refractory schizophrenia, as demonstrated in this study, exemplifies the clinical challenges associated with accurately interpreting the nature of hallucinatory experiences.

Moving forward, such findings strongly advocate for further investigative research to delve deeper into the potential of HHEs and SP in exacerbating pre-existing psychopathology. Subsequent research endeavors should explore the impact of these sleep-related experiences on various neuro-psychiatric disorders, shedding light on potential causal relationships and therapeutic implications. This avenue of research not only holds promise for advancing our understanding of the interplay between sleep neurobiology and psychiatric disorders, but also paves the way for targeted therapeutic interventions. Such advancements have the potential to revolutionize clinical practice, offering bespoke interventions to mitigate the impact of sleep-wake disruptions on vulnerable populations, and spearheading innovative approaches to the treatment of psychiatric disorders.
