# Correction: A Dynamic Foundation: Aberrations of Sleep Architecture and Its Association With Clinical and Sub-clinical Psychopathology

**DOI:** 10.7759/cureus.c346

**Published:** 2025-10-03

**Authors:** Richard C Todd

**Affiliations:** 1 Psychiatry, International European University School of Medicine, Kiev, UKR; 2 Research, Trinity Medical Sciences University, Warner Robins, USA

This article has been corrected to fix the following typos in the paragraph immediately preceding Figure 1: "Overall, 82 report records were **screened**, with 51 being excluded, as depicted in Figure 1 below." has been corrected to "Overall, 82 report records were **identified**, **76 were screened**, with 51 being excluded, as depicted in Figure 1 below."

